# A brief history of *MECP2* duplication syndrome: 20-years of clinical understanding

**DOI:** 10.1186/s13023-022-02278-w

**Published:** 2022-03-21

**Authors:** Daniel Ta, Jenny Downs, Gareth Baynam, Andrew Wilson, Peter Richmond, Helen Leonard

**Affiliations:** 1 Telethon Kids Institute, University of Western Australia, Perth, WA Australia; 2 Curtin School of Allied Health, Curtin University, Perth, WA Australia; 3 WA Department of Health, Genetic Services of Western Australia, Subiaco, WA 6008 Australia; 4 Western Australian Register of Developmental Anomalies, King Edward Memorial Hospital, PO Box 134, Subiaco, WA 6904 Australia; 5 Northern Entrance, Perth Children’s Hospital, 15 Hospital Ave, Nedlands, WA 6009 Australia; 6 Discipline of Paediatrics, School of Medicine, University of Western Australia, Perth, WA 6009 Australia

## Abstract

*MECP2* duplication syndrome (MDS) is a rare, X-linked, neurodevelopmental disorder caused by a duplication of the methyl-CpG-binding protein 2 (*MECP2*) gene—a gene in which loss-of-function mutations lead to Rett syndrome (RTT). MDS has an estimated live birth prevalence in males of 1/150,000. The key features of MDS include intellectual disability, developmental delay, hypotonia, seizures, recurrent respiratory infections, gastrointestinal problems, behavioural features of autism and dysmorphic features—although these comorbidities are not yet understood with sufficient granularity. This review has covered the past two decades of MDS case studies and series since the discovery of the disorder in 1999. After comprehensively reviewing the reported characteristics, this review has identified areas of limited knowledge that we recommend may be addressed by better phenotyping this disorder through an international data collection. This endeavour would also serve to delineate the clinical overlap between MDS and RTT.

## Introduction

The clinical understanding of *MECP2* duplication syndrome (MDS; OMIM 300260) has been limited both by its rarity and the short history of its recognition as a distinct disorder. Although the syndromic phenotype was described in 1999 by Lubs and colleagues [1], it wasn’t until 2005 that duplication of the *MECP2* gene was reported to be a cause of intellectual disability (ID) in the first case series on the disorder [2]. Moreover, the term ‘*MECP2* duplication syndrome’ was only coined in 2009 [3, 4]. There is a lack of epidemiological studies on MDS, with only a single Australian study estimating the birth prevalence to be 0.65/100,000 (1/150,000) live births and 1/100,000 for males (although it is likely that this is an underestimate of the true prevalence) [5]. In just over 20-years of case studies/series, the MDS phenotype has been documented to consist of ID, developmental delay, hypotonia, predisposition to infections, epilepsy, gastrointestinal (GI) issues and dysmorphic features as well as a variety of other comorbidities [1–87].

Earlier studies on the functional disomy of larger, cytogenetically-visible Xq28 duplications including the *MECP2* gene, documented characteristics such as microcephaly, an abnormal palate and hypoplastic genitalia, some of which may be attributable to other genes [11]. As such, this review will focus on submicroscopic duplications involving the *MECP2* gene which may include clinically relevant nearby genes such as *IRAK1*, *L1CAM* and *RAB39B*. This review will document the 20-year history of research publications that have contributed to the current clinical understanding of patients with MDS and carrier females, as well as the biological function and relevance of the *MECP2* gene in disease.

## Role of the *MECP2* gene in function and disease

### Biological function of *MECP2*

**Fig. 1:**
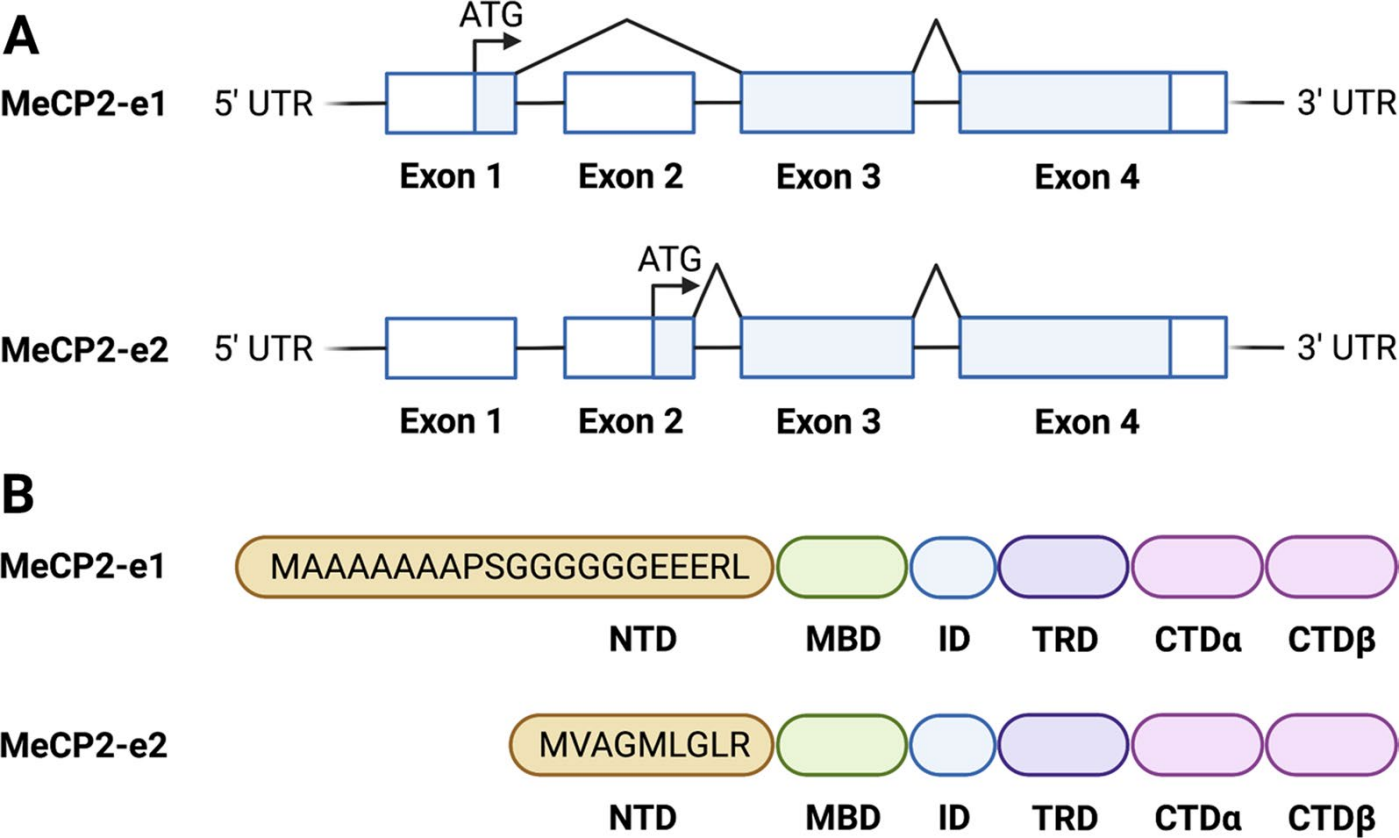
**A** The human *MECP2* gene is composed of four exons which can be alternatively spliced to produce two transcripts: *MECP2_e1* and *MECP2_e2*. **B** The former transcript skipping exon 2 with translation initiation in exon 1 encodes MeCP2-e1, which is longer with 498 amino acids and 21 unique N-terminal domain (NTD) amino acids. The latter transcript skipping exon 1 with translation initiation in exon 2 encodes MeCP-e2, which is shorter with 486 amino acids and 9 unique NTD amino acids. The remaining sequence both protein isoforms are identical, containing a methyl-CpG-binding domain (MBD), interdomain (ID), transcriptional repression domain (TRD) and C-terminal domain (CTD). Created with BioRender.com

Methyl-CpG-binding protein 2 (MeCP2) is a nuclear protein encoded by the *MECP2* gene (OMIM 300005) located on the long (q) arm of the X chromosome (Xq28). MeCP2 is a ubiquitous protein but is most highly expressed within postnatal neurons in the brain [88], increasing postnatally with age and neurogenesis [89]. In 1992, MeCP2 was discovered by Dr Adrian Bird as a transcriptional repressor of gene expression which binds to symmetrically, methylated cytosine-guanine (5’-CpG-3’) dinucleotides in DNA to alter chromatin structure [90]. *MECP2* contains four exons that encodes for two isoforms: MeCP2-E1 and MeCP2-E2 (Fig. 1). The transcript skipping exon 2 and that has translation initiation in exon 1 encodes for MeCP2-E1 and is slightly longer (498 amino acids) with 21 unique N-terminal amino acids, whilst the transcript containing all exons initiates translation in exon 2 to encode for MeCP2-E2 (486 amino acids) with 9 unique N-terminal amino acids [91]. Apart from the N-terminal domain (NTD), both MeCP2 isoforms are identical and have similar yet unique interacting protein partners and have shared as well as specific regulation of different genes [92]. Both MeCP2 isoforms contain important conserved regions such as the methyl-CpG-binding domain (MBD) for chromosomal localisation, an interdomain (ID), a transcriptional repression domain (TRD) responsible for recruiting the Sin3a repressor complex including histone deacetylases, a nuclear localization signal (NLS) which acts as a motif which tags proteins mediating transport to the nucleus and a C-terminal domain (CTD) [93–95].

DNA methylation is an epigenetic mechanism to repress gene transcription by the transfer of a methyl group by DNA methyltransferases to a cytosine in DNA to form 5-methylcytosine (5-mC) [96]. This process occurs mostly at cytosines preceding a guanine nucleotide (CpG) and can repress gene expression by directly preventing the association of transcription factors (TFs) to methylated promoters [97], or indirectly by competing with TFs at methylated CpG sites and altering chromatin structure via transcriptional repressors that recognise CpG sites [94]. MeCP2 is capable of recognizing such DNA and histone methylation marks and act as methylation dependent transcriptional modulator, in both a repressive and activating manner [98, 99]. Other proposed roles of MeCP2 include chromatin regulation [100] and RNA processing [101, 102], but the precise biological function and interactions of MeCP2 remains unclear and require further elucidation.

### Role of *MECP2* in disease: Rett syndrome

Prior to the association with MDS, *MECP2* was associated with Rett syndrome (RTT; OMIM 312,750). First described in 1966 by Andreas Rett [103], and thereafter by Hagberg and colleagues in 1983 [104], RTT is a neurodevelopmental disorder found primarily in females with a birth prevalence of 1/9,000 females [105]. *MECP2* gene mutations such as Arg133Cys, Thr158Met and Arg106Trp in RTT were specifically detected in 1999 by Huda Zoghbi and colleagues [106], with subsequent identification of further common missense, nonsense and deleterious mutations associated with RTT [107]. The clinical signs for RTT include four main criteria: partial or complete loss of (1) acquired purposeful hand skills and (2) acquired spoken language, (3) gait abnormalities (dyspraxic or absence of ability), and (4) hand stereotypies [108]. Individuals with RTT may experience 6–18 months of normal development before the onset of the aforementioned regression as well as intellectual disability, seizures, altered breathing patterns (hyperventilation and/or breath holding) and autistic features such as social withdrawal [107]. Most individuals with RTT are female due to the protective effect of X-inactivation, whereas most males with a RTT-causing mutation die prenatally or in the first few years of life due to congenital encephalopathy [109].

The intersection of MDS and RTT is best described as a ‘Goldilocks paradigm’ [110], in which too much of MeCP2 (MDS) and too little (RTT) will result in a severe disease state and highlights the importance of regulating this multi-functional protein and the difficult task of potential therapeutic strategies in maintaining a narrow range of *MECP2* levels. To date, no study has extensively compared the medical comorbidities between RTT and MDS.

## Evidence of a new X-linked intellectual disability syndrome: *MECP2* duplication syndrome

In 1999, Lubs and colleagues reported a family with 5 affected males who all shared X-linked severe intellectual disability (XLID), mild to moderate hypotonia, gastro-oesophageal reflux, swallowing dysfunction and recurrent respiratory infections [1]. Facial dysmorphisms including down slanting palpebral fissures, hypertelorism and a short nose with a depressed nasal bridge were also present. Linkage analysis localized the causal gene to the terminal 5 cM region of the Xq28 band. While numerous XLID disorders are mapped to this region [111], the phenotype described in this study was distinguished by recurrent respiratory infections and was initially referred to as ‘Lubs X-linked mental retardation syndrome’. While this is now known as ‘*MECP2* duplication syndrome’, the original nomenclature was still used as recently as 2010 [25].

Preceding the breakthrough studies in the mid-to-late 2000s, smaller studies highlighted the clinical similarities between MDS and RTT. From 2004 to 2005, two studies using real-time quantitative PCR to detect rearrangements in *MECP2* found a previously undetected *MECP2* duplication in: (1) a 34-year old female diagnosed with the preserved speech variant of Rett syndrome [10], and (2) an 8-year old boy [12]. The woman could walk unassisted and speak in short sentences until 9-years of age when she developed seizures that progressed to drug-resistant generalized tonic–clonic and atonic seizures resulting in regression of communication- and motor-skills. Later, she became hypotonic, could not walk, speak, or use precise hand movements and displayed hand-washing stereotypies. In contrast, the boy had severe intellectual disability, hypotonia, delayed psychomotor development, hand stereotypies, loss of purposeful hand use and could only babble at 6-years old. At the same age, drug-resistant myotonic-astatic and tonic seizures developed, which were subsequently followed by further regression of motor abilities and basic communication skills and increasing swallowing problems and bruxism. Such characteristics are now known to be commonly shared between RTT and MDS; these two early studies highlight the clinical similarities between both disorders.

In 2005, Van Esch and colleagues documented four families with a history of XLID and using array comparative genomic hybridization (array-CGH) and quantitative PCR (qPCR), found 13 males with a < 450 kb duplication of the Xq28 region including *MECP2* exhibiting a form of intellectual disability associated with progressive spasticity [2]. All the males were found to have facial hypotonia and greater lower limb spasticity while 10/12 had absent speech, 7/12 never walked, 5/9 had severe respiratory infections and 6/11 died before 25 years of age. Of interest, the ID-related L1 cell adhesion molecule (*L1CAM*) gene was also duplicated in these individuals and until this point no small duplications involving only *L1CAM* had been reported. Thus, it was not known if the *L1CAM* gene was associated with the MDS phenotype. However, the duplicated region in the previously reported case study of an 8-year-old boy [12] was located 5’ of *L1CAM* which left the gene intact. This suggests that the critical duplicated region harbors *MECP2* as the only ID-related gene.

Genotype–phenotype association studies since have shown that the minimally duplicated critical region for the MDS core phenotype includes *MECP2* and the interleukin-1 receptor associated kinase 1 gene (*IRAK1*; OMIM 300283) [4, 14, 19, 25, 28, 30, 40, 42, 44, 49, 52, 58, 112]. As the first major case series to detail the occurrence of *MECP2* duplication in males with severe intellectual disability [2], this study further refined the distinct phenotype identified in previous case studies and was followed by a rapid series of observational studies with larger patient samples.

Female carriers are defined as females who harbour a duplication of the *MECP2* gene but do not express the cardinal features of MDS. Whilst most female carriers are asymptomatic often due to skewed inactivation [2, 12–14, 16, 18, 20, 22, 25, 28, 36, 38, 52, 55, 56, 63, 67, 69, 75, 83, 86], some have been reported to manifest neuropsychiatric symptoms, learning disabilities and/or health issues [3, 4, 42, 43, 68, 73]. The seminal case series documented by Ramocki and colleagues in 2009 [4] was the first to describe the clinical features of nine female carriers (range 34–64 years). Five of these women experienced abnormal menses (56%), with four experiencing lifelong irregular menstrual cycles (three experienced premature menopause before the age of 40 and the fourth clinical symptoms of impending premature menopause with hot flashes and hair/skin changes). Endocrine and autoimmune disorders were also present in these women with two having type-2 diabetes and a third being pre-diabetic. Four had hypothyroidism and one Sjogren syndrome and fibromyalgia. Of the eight women that completed psychological evaluation and the Symptom Checklist 90-R, half were treated for depression prior to the birth of a child with MDS. All eight women exhibited symptoms of anxiety and for six, anxiety was present prior to the birth of the affected child. All the women endorsed compulsive behaviours such as the need for structure, routine, cleanliness, and order. Of the seven women who completed the Broad Autism Phenotype Questionnaire, elevated scores were seen for rigid personality (n = 7), pragmatic language deficits (n = 4) and aloof personality (n = 3) while four subjects exceeded the cut-off score for the broad autism phenotype. X-chromosome inactivation (XCI) studies showed that eight of the nine females had nearly 100% X-inactivation ratio (as one patient’s sample was non-informative).

Through available genetic testing reports, approximately four-fifths of *MECP2* duplications have been shown to be inherited, mainly maternal [1–5, 8, 9, 12, 14–20, 22–25, 28, 32, 34, 36–40, 42–44, 46, 48, 52, 53, 55, 56, 61, 63–65, 67–69, 73, 75, 77–79, 81, 83, 86], and one-fifth to arise de novo [5, 9, 11, 16, 17, 21, 23, 26, 27, 29, 32, 33, 43, 45, 47, 54, 56, 65, 70, 74, 77–79, 81, 83]. Paternally inherited *MECP2* duplications are less common [7, 35, 41, 55, 59, 70, 74].

These studies have allowed for the distinction of a clinically recognizable disorder, but further phenotyping will be required.

## The syndromic phenotype of *MECP2* duplication syndrome

### Early development and communication skills (Table 1)

**Table 1:** Summary of communication skills, gross motor function and neurological signs found in patients with MDS reported in the literature; NR = not reported

Feature	Males n/N	Females n/N	Total n/N (%)	References
**Communication skills**				
Absent speech	174/231	6/31	180/262 (69%)	[2, 3, 5, 7, 9, 11, 13, 14, 16, 18, 19, 21, 22, 25, 26, 29–35, 38, 41–44, 46–48, 51–55, 57, 59, 63, 64, 70, 73, 74, 77, 84–86]
Few words/limited speech	48/228	19/35	67/263 (25%)	[2, 3, 5, 7, 9, 12, 13, 16, 18, 21, 22, 25, 26, 29, 31–35, 37, 38, 41–44, 46–48, 51–55, 57, 59, 63, 64, 73, 74, 76, 77, 84]
**Gross motor function**				
Acquisition of head control	34/37	0/1	34/38 (89%)	[24, 31, 38, 47, 48, 68, 75, 84]
Acquisition of sitting	94/102	9/10	103/112 (92%)	[21, 22, 24, 26, 28, 29, 31, 36, 38, 47, 48, 59, 63, 64, 74–76, 84]
Acquisition of walking	171/265	13/20	184/285 (65%)	[3, 10, 14–19, 21, 22, 24–26, 28, 30–32, 36, 38, 42, 44, 46–48, 51–54, 57, 59, 63, 64, 68, 70, 73–77, 84, 85]
**Neurological signs**				
Ataxia or ataxic/wide-based gait	66/109	10/23	76/132 (58%)	[3, 5, 19, 20, 25, 26, 38, 43, 44, 46, 47, 52–55, 61, 70, 74, 75, 77]
Spasticity	86/187	4/13	90/200 (45%)	[1, 2, 4, 8, 13, 16–20, 25, 30, 35, 38, 41, 43, 46, 51–54, 60, 64, 69, 71, 73, 83–86]
Choreiform movements	16/24	1/3	17/27 (63%)	[4, 5, 31, 73]

Neonatal complications include early infantile hypotonia (388 of 441 individuals from 62 studies [1, 3, 4, 7, 9–27, 29–34, 36, 38, 41–43, 46–48, 51–53, 55, 59–61, 63, 64, 67, 69–71, 73–78, 81–86]), feeding difficulties with poor sucking [16, 73, 77], bowel obstruction [17, 74], malaise and vomiting [77], respiratory distress [77], breathing problems [84], hospitalisation for infections [77, 84] and failure to thrive [1, 9, 12, 13, 16, 17, 22, 25, 27, 29, 32, 38, 43, 44, 51, 61, 64, 67, 81, 82]. Developmental/psychomotor delay has been reported in most (n = 324) of 343 individuals from 56 studies [8, 11, 12, 14–16, 19–21, 23–27, 29, 31–33, 35–39, 41–44, 46–48, 51–55, 57, 59–61, 63, 64, 66–70, 72–77, 79, 82, 84, 86], leading to moderate to severe intellectual disability (280 of 286 individuals from 57 studies [1, 2, 4, 7–9, 11–23, 25–35, 37–40, 42, 43, 46–48, 52–56, 59, 64, 68–71, 73, 75, 80, 83–85]). Most children have impaired communication skills, as more than two-thirds (n = 179) of 261 individuals from 46 studies [2, 3, 5, 7, 9, 11, 13, 14, 16, 18, 19, 21, 22, 25, 26, 29–35, 38, 41–44, 46–48, 51–55, 57, 59, 63, 64, 70, 73, 74, 77, 84, 85] did not develop speech and a quarter (n = 67) of 263 individuals from 42 studies [2, 3, 5, 7, 9, 12, 13, 16, 18, 21, 22, 25, 26, 29, 31–35, 37, 38, 41–44, 46–48, 51–55, 57, 59, 63, 64, 73, 74, 76, 77, 84] were reported to have limited speech with the use of single words of simple phrases (Table 1). Speech delay was evident in almost all children [8, 15, 21, 22, 28, 31–33, 35, 40, 42–44, 47, 51, 55, 57, 63, 67, 74–76, 83]. Some individuals have been shown to develop better communication strategies using communication devices or aids [77].

### Gross motor function and neurological features (Table 1)

Difficulties in development of gross motor skills are apparent. The acquisition of head control was observed in most (n = 34) of 38 individuals across eight studies, as well as the acquisition of sitting (103/112 individuals across 18 studies; Table 1). In our earlier case series, over 90% (45/49 males and 7/7 females) acquired independent sitting—a half of males learned to sit by 12 months (range 3.5–36 months) and half of females by 15 months (range 6–19 months) [76]. However, walking was acquired in approximately two-thirds (n = 184) of 285 individuals observed in 41 studies (Table 1). In our case series, we estimated a 25% likelihood of achieving independent walking by 4 years for males and 2 years for females using time-to-event analysis [76].

Prominent neurological features have also been reported including ataxia or a wide-based gait in three-fifths (n = 76) of 132 individuals from 20 studies, spasticity (often lower-limb) in under a half (n = 90) of 200 individuals from 30 studies, and choreiform movements in 17 of 27 individuals from four studies (Table 1). Less commonly reported issues include abnormal deep tendon reflexes (chiefly hyperreflexia) [2, 8, 16, 19, 25, 28, 48, 53, 61], dyskinesia [19, 28], hyperkinesis [31, 70], and upper motor neuron syndrome (pyramidal syndrome) [19, 31].

### Developmental and/or intellectual regression (Table 2)

**Table 2:** Summary of regression found in patients with MDS reported in the literature; NR = not reported

Feature	Males n/N	Females n/N	Total n/N (%)	References
Developmental and/or intellectual regression	–	–	110/268 (41%)	[3, 4, 8, 10, 12, 36, 38, 41, 43, 48, 52, 53, 57, 61, 68, 71, 75, 77–80, 82–84, 113]
Regression of speech/ communication skills	8/17	5/8	13/25 (52%)	[3, 4, 10, 36, 38, 41, 43, 53, 68]
Regression of gross motor skills	–	–	36/93 (39%)	[3–5, 10, 12, 36, 38, 43, 53, 57, 61, 76, 77, 80]
Regression of purposeful hand use	13/59	1/8	14/67 (21%)	[4, 10, 12, 76]

Developmental and/or intellectual regression has been reported in two-fifths (n = 110) of 268 individuals in 24 studies (Table 2). A subset of such studies has differentiated between the loss of particular skills, with regression of speech/communication skills occurring in a half (n = 13) of 25 individuals across 10 case studies, regression of gross motor skills (mainly walking) occurring in two-fifths (n = 36) of 69 individuals across 14 studies, and regression of purposeful hand use occurring in one-fifth (n = 14) of 67 individuals across four studies (Table 2). The onset of regression has been observed in parallel with seizure occurrence or exacerbation. In two recent large case series affecting 20/22 and 12/12 individuals respectively, regression was linked to onset or progression of treatment-resistant epilepsy and only a few individuals regressed spontaneously [77, 79]. Loss of ambulation has also been described following a pneumococcal chest infection [28] Whilst epilepsy and possibly recurrent infection may be contributing factors to regression, further understanding of the timing and association of events with regression, the possible causes and types of skills lost would be valuable.

### Epilepsy (Table 3)

**Table 3:** Summary of epileptic features found in patients with MDS reported in the literature and proportion of seizure types in individuals with seizures from prominent case series investigating epilepsy; NR = not reported

Feature	Males n/N	Females n/N	Total n/N (%)	References
Epilepsy/seizures	–	–	326/619 (53%)	[1–4, 7–23, 25–42, 44–48, 51–55, 57–61, 63, 64, 66–71, 73–80, 82–86, 113]
Treatment-refractory seizures	–	–	100/148 (68%)	[1, 10, 12, 18–20, 28, 33, 37, 40, 41, 43, 44, 51–53, 57, 60, 61, 70, 73, 74, 76, 77, 79, 80]

Seizure type	Miguet et al. [77]	Marafi et al. [79]*	Cutri-French et al. [113]*
	n/N (%)	n/N (%)	n/N (%)
**Generalised**			
Tonic–clonic	19/35 (54%)	15/22 (68%)	11/22 (50%)
Tonic	NR	14/22 (63%)	NR
Myoclonic	8/35 (23%)	14/22 (63%)	4/22 (18%)
Absence	5/35 (14%)	NR	5/22 (23%)
Atypical	NR	14/22 (63%)	NR
Atonic	12/35 (34%)	18/22 (82%)	7/22 (32%)
**Focal**			
Focal	NR	8/22 (36%)	1/22 (5%)
Complex	6/35 (17%)	NR	6/22 (27%)

Other	Total n/N (%)		References	
Lennox-Gastaut syndrome	17/49 (35%)		[64, 79, 84]	

*A proportion of individuals may have been documented in both these case series

The development of epilepsy/seizures is one of the dominant neurological morbidities in MDS, appearing in over a half (n = 326) of 619 individuals from 76 studies (Table 3). Time-to-event analysis in a recent case series [76] of 56 individuals found the risk of developing seizures was 53% by the age of 9 years, suggesting that seizure onset may be later than in other developmental encephalopathies. Smaller case series have reported a median seizure onset of 4.4 years (range 0.2–22 years) in 16/24 individuals [84], and 5.3 years (IQR: 2 years–10 years) in 22/49 individuals [113], to 9 years (range 1–20 years) in 24/55 individuals [76], or a mean onset being 6 years (range 1 day–19 years) in 22/47 individuals [79], and 7.4 years (range 0.4–35 years) in 35/59 individuals [77].

Recent large case series attempting to characterise the epileptic phenotype of MDS have documented different proportions of seizure types amongst individuals (Table 5) [77, 79, 113], however the frequency of certain seizure types remains unclear and sample sizes from recent case series remain small. Seizure types include a) generalized seizures: tonic–clonic, tonic, myoclonic, absence, atypical absence, atonic and b) focal and complex focal seizures [84, 113]; with atonic seizures possibly necessitating wheelchair use [57], Severe epileptic encephalopathy represented by Lennox-Gastaut syndrome (LGS) has been reported in 17/49 individuals from three studies [64, 79, 84]. Seizures are often intractable. In two-thirds (n = 100) of 148 individuals from 26 studies (Table 3) there was difficulty in reducing the frequency and intensity of seizure activity, despite the use of multiple antiseizure medications (ASMs) [84]. The most commonly-used ASMs include valproic acid and levetiracetam, but no specific monotherapy or polytherapy with a sustained effect on seizure control has been identified [79, 84].

Adjuvant therapies such as vagus nerve stimulation (VNS) have been discussed in only two recent case series where six individuals were reported to receive VNS treatment [79, 84]. Whilst there was no comment on the efficacy in one individual [84], four of the remaining five were reported to experience reduced overall seizure frequency, severity and duration with a particular improvement in atonic seizures [79]. The ketogenic diet or a modified Atkins diet has also been reported to be used by eight individuals of whom six were reported to have modest improvements in seizure frequency and severity [44, 79]. Deep brain stimulation has been reported in one case study of a 35-year-old male individual [57]. At the age of 20 years, the individual received implantation of DBS electrodes and pulse generator for stimulation of the anterior nucleus of the thalamus (AN-DBS); and was treated in conjunction with carbamazepine and vigabatrin from 3-months prior to 2-years post-surgery. The individual’s generalized tonic–clonic seizures decreased in frequency from 125/month prior to implantation to 60/month and 45.7/month one-year and four-years post-stimulation respectively; complex partial seizures also decreased from 4–5/day to 2–3/week at 2-years follow up. Corpus callosotomy has also been undertaken in a few individuals [64, 80], with improved seizure control and reduction in use of ASMs experienced in one boy who also subsequently regained previously lost walking and vocalization abilities [80]. With the detrimental effect of seizures in MDS, further systematic evaluation of treatment responsiveness and epilepsy frequency/types is needed.

### Respiratory health (Tables 4 and 5)

Beyond recurrent infections, other respiratory comorbidities contribute to MDS morbidity. Non-specific breathing disturbances have been reported in 30 of 98 individuals reported from nine different studies (Table 4). Congenital and early childhood symptomatology include subglottic stenosis [40], bronchomalacia (2/2) [24, 32], laryngomalacia/ pharyngomalacia (10/23) [11, 32, 40, 43, 71, 82], and tracheomalacia (4/21) [11, 14, 32, 40]. Later onset symptomatology such as breathlessness [5], chronic coughing/wheezing (4/9) and bronchospasm [13, 82], may be related to diagnoses of asthma (7/35), reactive airway disease [40], bronchiectasis [81], or upper airway obstruction [64]. Resultant hypoxemia may occur [72], and as such individuals may require oxygen supplementation [82], or ventilatory support [13]. Respiratory infections such as pneumonia, bronchitis and bronchiolitis have been reported in almost three-quarters (n = 367) of 498 individuals from 60 studies (Table 5) [1–4, 8–10, 12–14, 16–22, 24–32, 34–38, 40–48, 51–53, 55–57, 59–64, 66–74, 76–78, 81–86], often resulting in frequent hospitalisation [8, 17, 64, 73, 76, 77, 81, 84]. Antibiotic therapy is commonly required to manage respiratory infections [72, 81], with severe presentations often resulting in multi-disciplinary management including immunoglobulin replacement [72], airway clearance therapy/devices [72, 78], and ventilatory support [28, 52, 57, 64, 76]. Treatment for respiratory tract secretions includes reducing the build-up of mucus by using techniques such as autogenic drainage, manually assisted cough, respiratory physiotherapy and the use of a positive expiratory pressure (PEP)-mask [81].

**Table 4:** Summary of respiratory comorbidities found in patients with MDS reported in the literature, excluding respiratory infections; NR = not reported

Feature	Males n/N	Females n/N	Total n/N (%)	References
Asthma	–	–	7/35 (20%)	[5, 18, 32, 40, 72]
Breathing problems	–	–	30/98 (31%)	[4, 5, 14, 37, 43, 54, 67, 71, 78]
Chronic coughing/wheezing	3/5	1/4	4/9 (44%)	[43, 72, 81, 82]
Pulmonary hypertension	–	–	5/111 (5%)	[5, 65, 77]
Bronchomalacia	2/2	NR	2/2 (100%)	[24, 32]
Laryngomalacia/pharyngomalacia	7/19	3/4	10/23 (43%)	[11, 32, 40, 43, 71, 82]
Tracheomalacia	4/21	NR	4/21 (19%)	[11, 14, 32, 40, 82]

**Table 5:** Summary of infections and immunopathology found in patients with MDS reported in the literature; NR = not reported

Feature	Males n/N	Females n/N	Total n/N (%)	References
Respiratory infections	–	–	367/498 (74%)	[1–4, 8–10, 12–14, 16–22, 24–32, 34–38, 40–48, 51–53, 55–57, 59–64, 66–74, 76–78, 81–86]
Pharyngitis	4/46	0/2	4/48 (8%)	[18, 62, 84]
Tonsilitis	4/24	6/12	10/36 (28%)	[18, 43, 62, 74]
Otitis media	–	–	23/77 (30%)	[5, 8, 9, 18, 40, 43, 74, 84, 85]
Urinary tract infections	–	–	14/56 (25%)	[11, 43, 51, 62, 84]
Sepsis	–	–	11/88 (13%)	[1, 5, 59, 62, 64, 68, 84]
Lymphadenopathy	2/8	NR	2/8 (25%)	[1, 64]

As well as for nutrition and hydration, gastrostomy has been used to reduce the risk of aspiration pneumonia due to swallowing problems (Table 7) [72, 84], which can lead to respiratory failure [52], and death [8]. Pulmonary hypertension has been reported in 5/107 (5%) patients and as a cause of death in four, all before three years of age [5, 65, 77]. It is likely that the high respiratory disease burden in MDS is associated with multiple hospital admissions, constant supportive care and interventional procedures that are underreported in the literature and require better characterization. Central hypoventilation syndrome has been reported in two individuals [24, 81]. It is a rare autonomic nervous system disorder defined as a reduced ventilatory response to hypercapnia and hypoxemia in the absence of pulmonary, cardiovascular or neuromuscular anomalies [114]. More generally, autonomic disturbances may be underreported and require further study.

### Other infections (Table 5)

Susceptibility to infections is a common feature in MDS, often manifesting in recurrent episodes that can appear more frequently in the first few years of life [8, 18, 22, 31, 35, 43, 44, 51, 52, 56, 62, 70, 76]. Ear infections have been reported in just under one-third (23/77) of patients, tonsillitis in just over one-quarter (10/36) and pharyngitis in 4 of 48 patients across 10 studies (Table 5). Conductive hearing loss can occur due to otitis media [9, 77], and may necessitate insertion of myringotomy tubes [9, 32, 67], or hearing-aid placement [32]. Urinary tract infections have also been detected in 14/56 individuals. All of these infections can result in sepsis, which has been reported in 11/88 (13%) of individuals and has even caused septic shock [84], or toxic shock syndrome [62].

### Congenital heart disease (Table 6)

**Table 6:** Summary of cardiovascular problems found in patients with MDS reported in the literature; NR = not reported

Feature	Males n/N	Females n/N	Total n/N (%)	References
**Septal defects**				
Atrial*	5/78	1/1	6/79 (8%)	[9, 32, 69, 77]
Ventricular	1/1	NR	1/1 (100%)	[67]
**Vascular defects**				
Patent ductus arteriosus	9/46	1/1	10/47 (21%)	[9, 17, 24, 32, 40, 68]
**Other**				
Cardiomegaly	4/87	NR	4/87 (5%)	[1, 5, 77]
Heart failure	5/21	NR	5/21 (24%)	[17, 48]
Myocarditis/pericarditis	1/5	1/1	2/6 (33%)	[1, 75]

*Atrial septal defect (ASD) and patent foramen ovale (PFO)

Vascular defects such as patent ductus arteriosus has been reported in 10 out of 47 patients in six studies and patent foramen ovale has been reported in 3 out of 20 patients in three studies [9, 32, 69] (Table 6). Less commonly reported have been atrial septal defects (6/79), ventricular septal defects [67], valvular heart disease such as aortic valvar stenosis [40], aortic root dilation [14], bicuspid aortic valve [17, 40], coarctation of the aorta [13, 40], and pulmonary stenosis [5]. Congenital heart disease (CHD) may be an underreported phenomenon in MDS [115]. When MDS is diagnosed neonatally [45] or in infancy, children should have echocardiograms to screen for CHD. As such, further attention to the cardiovascular profile of patients with MDS is required in future studies.

### Gastrointestinal problems (Table 7)

Functional issues of the gastrointestinal system are a major clinical problem in MDS (Table 7), with swallowing difficulties being reported in half (n = 60) of 119 individuals from 16 different studies. This has likely implications for feeding problems reported in half (n = 97) of 186 patients from 28 different studies and the risk of aspiration pneumonia. Commonly, nasogastric or percutaneous endoscopic gastrostomy (PEG) feeding has been utilised for feeding difficulties and to reduce the risk of aspiration pneumonia [19, 28, 43, 51, 52, 57, 58, 64, 81]. More than half (n = 141) of patients from 18 different studies (Table 7) have been reported to experience gastroesophageal reflux which can also contribute to respiratory morbidities such as bronchitis and pneumonia [76, 81, 85, 116], but is often treated with fundoplication [17, 32, 52, 58]. Around three-quarters (n = 330) of 456 patients from 36 different studies (Table 7) have presented with constipation. A high-fibre diet and enemas have been used to treat constipation [17, 67]. With the reporting of growth deficiency and failure to thrive in some patients, early surveillance of feeding issues, gastroesophageal reflux and constipation is important to minimize the risk of compromised growth and malnutrition [76]. Hirschsprung disease, a neurocristopathy characterised by the variable absence of enteric ganglion cells in the submucosal and myenteric plexus of the gastrointestinal tract, has been reported in two cases of MDS [22, 24]. This disorder can result in sustained contraction of the aganglionic bowel segment causing bowel obstruction, constipation and failure to thrive. Consideration of an intestinal/rectal biopsy may be important in infants with MDS, particularly in the presence of bowel obstruction, constipation and failure to thrive [22, 24, 117].

**Table 7:** Summary of gastrointestinal problems found in patients with MDS reported in the literature; NR = not reported

Feature	Males n/N	Females n/N	Total n/N (%)	References
Abdominal bloating	–	–	18/47 (38%)	[22, 76]
Bowel/pseudointestinal obstruction	4/25	NR	4/25 (16%)	[17, 38, 52]
Constipation	–	–	330/456 (72%)	[4, 11, 17, 18, 22, 24, 25, 27, 29, 30, 32, 35, 38, 40, 42, 43, 48, 51, 59, 63, 64, 67, 69–71, 74–78, 80, 83–87]
Drooling	–	–	118/168 (70%)	[4, 12, 13, 20, 21, 25, 27, 31, 32, 34, 43, 44, 52, 59, 71, 73, 77, 82, 86]
Swallowing difficulties	57/112	3/7	60/119 (50%)	[1, 3, 5, 12, 13, 19, 25, 28, 32, 38, 43, 51, 52, 59, 84, 85]
Aspiration	14/32	1/2	15/34 (44%)	[1, 3, 32, 40, 52, 53, 57, 64, 72, 81, 82]
Feeding problems	88/163	9/23	97/186 (52%)	[5, 9, 11, 16, 21, 25, 27, 29, 31, 32, 36, 43, 44, 51, 55, 59, 63, 64, 69–71, 73–75, 77, 81, 84, 86]
Gastro-oesophageal reflux	–	–	141/261 (54%)	[1, 4, 8, 11, 13, 16, 32, 38, 40, 43, 54, 64, 71, 76, 77, 81, 83–85]
Hirschsprung disease	2/4	NR	2/4 (50%)	[17, 22, 24]

“Functional abnormalities of the GI tract” have been reported in 11/20 (55%) individuals with MDS, [83] with no further description

### Musculoskeletal health

Scoliosis/kyphoscoliosis is the most commonly reported orthopaedic issue reported, affecting over a quarter of patients (58/211) patients in 14 studies [4, 29, 40, 43, 44, 53, 69, 70, 74, 76–78, 81, 84]. Time-to-event analysis in our case series estimated that approximately half of males would develop spinal curvature by 22 years of age [76]. Progressive scoliosis can be associated with restrictive lung disease and as such surveillance is important [53]. Contractures of the ankle, knees, hip/trunk, elbows, and wrist joints have also been reported [17, 19, 20, 25, 29, 48, 64, 77], likely caused by spasticity observed in MDS. It has been recommended that botulinum toxin be a considered treatment for contractures and to prevent joint dislocations [25, 30]. Less common musculoskeletal problems such as juvenile idiopathic arthritis [67], osteopenia/osteoporosis [66, 85], joint luxations/subluxations [40, 43], joint hypermobility [7, 26, 27, 55], muscular atrophy [1], lordosis [28], and torticollis [32, 40] have also been reported. Fractures have been reported [17, 32, 66, 77], however further research is required to confirm the prevalence and underlying mechanisms.

### Urogenital issues (Table 8)

**Table 8:** Summary of urogenital issues found in patients with MDS reported in the literature; NR = not reported, NA = not applicable

Feature	Males n/N	Females n/N	Total n/N (%)	References
**Urinary system**				
Vesicoureteral reflux	2/4	1/1	3/5 (60%)	[9, 35, 36]
Bladder dilation/hypertrophy	5/58	NR	5/58 (9%)	[25, 45, 77]
Duplex kidney	1/7	1/4	2/11 (18%)	[5, 14]
Hydronephrosis	6/70	NR	6/70 (9%)	[14, 36, 56, 64, 77]
Pyelonephritis	3/22	1/4	4/26 (15%)	[43, 62]
Renal stones	2/18	NR	2/18 (11%)	[36, 69]
Ureteral dilation	5/53	NR	5/53	[77]
**Genitals**				
Unilateral/bilateral cryptorchidism	50/140	NA	50/140 (36%)	[4, 5, 7–9, 12, 14, 16, 21, 22, 25, 29, 31, 32, 38, 51, 63, 69, 77, 85, 86]
Micropenis	12/77	NA	12/77 (16%)	[5, 40, 51, 77]
Hypospadias	3/13	NA	3/13 (23%)	[14, 46, 71]
Hypogenitalism	6/15	1/1	7/16 (44%)	[7, 9, 25, 29, 32]

The most commonly reported urogenital issue has been the presence of unilateral or bilateral cryptorchidism, identified in over a third of males (n = 50) in 140 patients in 20 studies, and which may require orchidopexy (Table 8) [8, 9, 25, 32, 51]. Other congenital anomalies in males include micropenis (12/77) and hypospadias (3/13). Bladder dilation/hypertrophy (5/58), hydronephrosis (6/70), pyelonephritis (4/26) and renal stones (2/18) may be associated with vesicourethral reflux (3/5), ureteral dilation (5/53) and urinary tract infections (Table 8). The presentation of gynecomastia reported in 7 of 16 patients also supports the existence of hypogenitalism as a clinical feature in this syndrome [77]. The exact prevalence of congenital anomalies of the kidney and urinary tract in MDS remains unknown but may become more apparent if more children undergo renal imaging.

### Symptoms of autism and behavioural features (Table 9)

**Table 9:** Summary of features associated with autism spectrum disorder (ASD) and other features of altered behaviour or mood found in patients with MDS reported in the literature; NR = not reported

Feature	Males n/N	Females n/N	Total n/N (%)	References
**Symptoms of autism**				
Autism diagnosis	31/46	3/4	34/50 (68%)	[4, 5, 39, 42, 49, 75]
Unspecified, general autistic features	24/44	5/9	29/53 (55%)	[12, 14, 20, 23, 25, 29, 31, 34–36, 38, 43, 52–54, 67, 68, 85]
Anxiety	–	–	44/111 (40%)	[4, 5, 26, 29, 42, 55, 69, 74, 78, 84]
Compulsive behaviours	1/5	2/3	3/8 (38%)	[5]
Difficulty adjusting to change	8/14	2/3	10/17 (59%)	[4, 5]
Gaze avoidance/difficulty using eye gaze	40/54	4/7	44/61 (72%)	[4, 5, 20, 25, 34, 41, 52, 57, 63, 67, 75, 83]
Hyperacusis	NR	2/5	2/5 (40%)	[41, 43]
Impaired social interactions	34/41	4/5	38/46 (83%)	[4, 26, 36, 38, 55, 67, 73, 75, 83]
Repetitive behaviours	18/23	3/7	21/30 (70%)	[4, 5, 10, 32, 36, 55]
Stereotypical behaviours	140/251	18/34	158/285 (55%)	[4, 5, 10, 12, 14, 17, 23, 25, 27, 29–34, 36–38, 40, 42, 43, 49, 53–55, 57, 59, 61, 63, 64, 69–71, 73–77, 83, 86]
**Other features of altered behaviour or mood**				
Aggression	NR	2/7	2/7 (29%)	[41, 74]
Attentional difficulties	1/1	6/8	7/9 (78%)	[26, 41, 55, 67, 70]
Bruxism	–	–	102/156 (65%)	[4, 5, 12, 32, 67, 71, 73, 77, 84]
Depression/depressive mood	1/1	1/3	2/4 (50%)	[41, 55]
High pain tolerance/pain insensitivity	–	–	102/171 (60%)	[4, 32, 77, 83, 147]
Hyperactivity	3/11	3/6	6/17 (35%)	[23, 49, 55, 70]
Night/inappropriate laughing	–	–	12/61 (20%)	[53, 74, 76]
Uncontrolled screaming spells	–	–	16/53 (30%)	[76]

The behavioural phenotype of MDS is diverse and can be associated with autistic features (Table 9). Autism was diagnosed in 34 of 50 cases in six studies [4, 5, 39, 42, 49, 75]. General autistic features that have not been validated with a clinical autism instrument have also been reported in other case series [12, 14, 20, 23, 25, 29, 31, 34, 35, 38, 43, 52–54, 67, 68, 85].

Prominent behavioural features include gaze avoidance in almost three-quarters of patients (44/61 [72%]), impaired social interactions (38/46 [83%]), teeth grinding (102/156 [65%]), and stereotypical movements in more than half (158/285 [55%]).Stereotypies such as finger sucking, biting and rubbing, midline hand movements, hand flapping, wringing, clasping, ‘knitting’, shaking, clapping, hand and arm waving, head movements such as bouncing down and to the side, vocal, rocking of the body, swinging of the upper part of the body and spinning [4, 27, 29–31, 33, 36, 37, 40, 42, 53, 57, 63, 70, 73, 75–77, 84] may present and persist, in variable ways, throughout life [77]. Hand or finger biting in particular can be self-injurious [4], particularly when pain insensitivity is a common characteristic.

As in RTT [118], decreased pain sensitivity has been reported in more than half of patients (102/171 [60%]) and can also be associated with temperature hyposensitivity [49, 83]. As a result, individuals may experience injuries such as fractures and express little to no pain [32, 77]. It has been suggested that an increase in *MECP2* copy number may be associated with the abnormal development of proprioceptive and nociceptive pathways [77].

### Dysmorphic features and presentations (Table 10)

**Table 10:** Summary of prominent dysmorphic features found in patients with MDS reported in the literature; NR = not reported

Feature	Males n/N	Females n/N	Total n/N (%)	References
**Head**				
Microcephaly (OFC < − 2 SD)	38/178	7/17	45/195 (23%)	[5, 7, 9, 11, 13, 14, 16, 19, 25, 27, 29, 31, 32, 38, 40, 43, 51, 69–71, 74, 77, 81, 84, 85]
Macrocephaly (OFC > + 2 SD)	25/146	4/23	29/169 (17%)	[1, 3, 5, 14, 19, 25, 32, 36, 38, 58, 65, 69, 71, 77, 85]
Brachycephaly	19/42	NR	19/42 (45%)	[14, 18, 19, 25, 32, 34, 40, 59, 73]
Plagiocephaly	14/44	NR	14/44 (32%)	[5, 7, 16, 18, 21, 25, 64, 85]
**Face/forehead**				
Facial hypotonia/hypomimic face	34/62	3/3	37/65 (57%)	[2, 10, 13, 14, 16, 25, 34, 51, 59, 71, 85]
Midface hypoplasia	67/99	NR	67/99 (68%)	[7, 14, 19, 25, 36, 40, 42, 59, 73, 77, 85, 86]
Long face	8/29	1/2	9/31 (29%)	[16, 21–23, 54, 69, 85]
**Hair**				
Sparse anteriorly	29/42	NR	29/42 (69%)	[77]
Thick and dense	39/43	1/6	40/49 (82%)	[40, 77]
**Eyes**				
Sparse eyebrows	24/47	1/4	25/51 (49%)	[17, 25, 43, 77]
Synophrys	15/62	2/3	17/65 (26%)	[16, 35, 38, 51, 77, 85]
Deep-set eyes	33/78	NR	33/78 (42%)	[14, 25, 77, 84, 86]
Downslanting palpebral fissures	12/33	2/2	14/35 (40%)	[1, 16, 21, 22, 38, 40, 51, 52, 59, 85]
Epicanthal folds	32/93	3/5	35/98 (36%)	[8, 11, 14, 16, 25, 38, 40, 43, 51, 67, 69, 77, 85]
Hypertelorism	48/121	6/10	54/131 (41%)	[1, 7, 9, 11, 14, 19, 25, 31, 32, 38, 51, 52, 55, 69, 70, 77]
Ptosis	18/63	2/5	20/68 (29%)	[14, 25, 27, 31, 38, 40, 43, 77]
Strabismus	50/72	1/1	51/73 (70%)	[21, 22, 25, 32, 48, 51, 68, 77]
**Ears**				
Large ears	82/131	1/2	83/133 (62%)	[7, 8, 14, 16, 19–21, 23, 25, 33, 36, 42, 47, 51, 59, 64, 69, 73, 77]
Low-set ears	15/31	2/4	17/35 (49%)	[16, 25, 32, 38, 51, 63, 69, 70, 73, 86]
**Nose**				
Short nose	14/29	1/1	15/30 (50%)	[1, 11, 16, 40, 51, 85]
Prominent tip of nose	29/43	1/1	30/44 (68%)	[52, 77]
Flat nasal bridge	17/31	3/6	20/37 (54%)	[25, 32, 38, 42, 43, 51, 52, 63, 73, 85]
Narrow nasal bridge	28/44	NR	28/44 (64%)	[77]
Prominent nasal bridge	48/95	NR	48/95 (51%)	[8, 14, 25, 32, 77, 84, 85]
Wide nasal bridge	21/40	4/8	25/48 (52%)	[11, 14, 16, 19, 25, 33, 43, 55, 85]
Upturned nares	28/65	NR	28/65 (43%)	[14, 25, 32, 63, 73, 77, 86]
**Mouth and lips**				
High-arched palate	17/51	3/6	20/57 (35%)	[7, 9, 14, 21, 22, 25, 29, 32, 38, 51, 53, 63, 70, 85]
Tented upper lip vermilion	18/41	2/4	20/45 (44%)	[32, 51, 69, 70]
Open mouth appearance	57/67	2/5	59/72 (82%)	[16, 25, 27, 31, 43, 59, 77, 85, 86]
Small mouth	64/117	3/5	67/122 (55%)	[8, 9, 11, 13, 16, 25, 32, 51, 63, 69, 70, 77]
Thick lower lip	37/48	1/1	38/49 (78%)	[18, 27, 77]
**Teeth**				
Teeth anomalies	32/34	NR	32/34 (94%)	[77]
Persistence of deciduous teeth	23/26	4/10	27/36 (75%)	[43, 74, 77]
Prominent central incisors	16/32	NR	16/32 (50%)	[77]
**Jaw**				
Micrognathia	11/35	4/6	15/41 (37%)	[7, 9, 11, 31, 32, 38, 40, 51, 52, 59, 64, 69, 70]
**Hands and feet**				
Clinodactyly	8/18	3/10	11/28 (40%)	[11, 14, 25, 32, 41, 65, 70]
Tapered fingers	55/80	NR	55/80 (69%)	[8, 11, 16, 77, 84, 85]
Small hands and/or feet	29/50	2/5	31/55 (56%)	[7, 11, 32, 48, 51, 70, 77]
Valgus flat feet	26/35	2/6	28/41 (68%)	[74, 77]

Dysmorphic features have been frequently reported in MDS with a wide variability of clinical presentations affecting various body structures (Table 10). Presentations can range from unnoticeable or mild to severe and pronounced, with the most comprehensive characterization of dysmorphic features provided in a French case series by Miguet et al. in 2018 [77]. Dysmorphic features change with age [77], and as such the prevalence of these characteristics are dependent on the relative age of patients between case series.

Although microcephaly and macrocephaly have been described in 45/195 and 29/169 patients respectively, it remains unclear whether abnormal occipitofrontal circumference (OFC) is a key clinical feature in MDS. Less commonly reported cranial dysmorphisms include brachycephaly (19/42) and plagiocephaly (15/44). Facial hypotonia, contributing to diminished facial expressions (hypomimia), has been reported in over a half of patients (37/65). Midface hypoplasia has been reported in two thirds of patients (67/99) and is a prominent clinical feature of MDS. Patients have also been described to have thick and dense hair (40/49).

Ocular features such as deep-set eyes (33/78), downslanting palpebral fissures (14/35) epicanthal folds (35/98) and hypertelorism (54/141) have also been commonly reported. Strabismus has been identified in a high proportion of patients (51/73), most frequently divergent strabismus (exotropia) [17, 25, 48, 77], which could be related to visual impairments such as farsightedness (hyperopia) [12, 25, 26, 74, 77], and amblyopia [40]. Large (83/133) and/or low-set (17/35) ears are common, as well as a narrow (28/44) and/or prominent (48/95) nasal bridge with upturned nares (28/65).

Many patients present with a small mouth (67/122) and an open mouth appearance (59/72). Up to three-quarters of patients have a persistence of deciduous teeth (27/36). Other reported presentations include an undersized jaw (micrognathia) (15/41) and anomalies of the extremities which include small hands and/or feet in over a half (31/55), tapered fingers (55/80) and pes planus (28/41).

No apparent differences have been detected in the proportion of such features between male and female patients, although there is insufficient data on the dysmorphology found in female patients with MDS and further investigation of the pathophysiology of the dysmorphic features in this disorder is needed.

### Autonomic problems (Table 11)

**Table 11:** Summary of autonomic problems found in patients with MDS reported in the literature; NR = not reported

Feature	Males n/N	Females n/N	Total n/N (%)	References
Breath holding	1/20	1/1	2/21 (10%)	[5, 27]
Episodes of hyperventilation	1/1	NR	1/1 (100%)	[46]
Episodes of hypoventilation	2/4	NR	2/4 (50%)	[24, 81]
Vasomotor troubles	–	–	58/100 (58%)	[77, 78, 84]
Livedo of the limbs	36/43	2/6	38/49 (78%)	[7, 11, 43, 77]
Cold hands and/or feet/problems regulating body temperature	4/15	NR	4/15 (33%)	[12, 18, 32]

Generalized vasomotor disturbances have been reported in more than a half of patients (58/100) in three studies (Table 11). Livedo or mottling of the extremities has also been found in over three-quarters (n = 38) of 49 patients from four studies [7, 11, 43, 77], although noted to be less apparent after adolescence in some [77]. Temperature dysregulation may have manifest as descriptions of cold hands and/or feet that appear pink/red with poor circulation [12, 17, 18, 78], hyperpyrexia or over-heating [28, 32], and shivering unrelated to infections [28]. Other reported signs consistent with possible dysautonomia have included occasional reports of recurrent episodes of breath holding for 2 of 21 patients from two studies [5, 27], and episodes of hyperventilation in just one patient [46] and hypoventilation in two others [24, 81].

### Sleep problems (Table 12)

**Table 12:** Summary of sleep problems found in patients with MDS reported in the literature; NR = not reported

Feature	Males n/N	Females n/N	Total n/N (%)	References
Sleep disturbances	–	–	62/112 (55%)	[4, 5, 33, 43, 54, 55, 71, 78, 84, 86]
Obstructive sleep apnoea	29/76	3/14	32/90 (36%)	[1, 5, 18, 43, 66, 74, 77, 81]
Hypersomnia/somnolence	17/93	NR	17/93 (18%)	[69, 76, 84]
Nocturnal awakening	5/22	NR	5/22 (23%)	[84]
Sleep–wake rhythm disorder	6/27	NR	6/27 (22%)	[1, 84]

Sleep disturbances have been reported in over a half of patients (62/112) in 10 studies; most frequently described as obstructive sleep apnoea, which was found in over a third (n = 32) of 90 patients from eight studies (Table 12). Ventilatory support for sleep apnoea can involve continuous positive airway pressure (CPAP) therapy and supplemental oxygen [81]. Surgical interventions such as adenoidectomy and/or tonsillectomy may be indicated for obstructive tissue such as enlarged adenoids or tonsils which can contribute to sleep apnoea [18, 77, 81, 86]. Hypersomnia/somnolence (17/93 from three studies), nocturnal awakening (5/22 from one study) and a sleep–wake rhythm disorder (6/27 from two studies) have been reported less frequently (Table 12).

### Neuroradiological findings (Table 13)

**Table 13:** Summary of prominent neuroradiological findings found in patients with MDS reported in prominent case series with MRI studies; NR = not reported

Seizure type	Honda et al. [85]	El Chehadeh et al. [65]	Takeguchi et al. [84]
	n/N (%)	n/N (%)	n/N (%)
**Abnormal imaging findings**	10/12 (83%)	28/30 (93%)	20/23 (87%)
≥ 2 brain MRI abnormalities	NR	25/30 (83%)	10/23 (43%)
Abnormal intensities in deep white matter	NR	6/30 (20%)	9/23 (39%)
Reduced white matter volume	3/11 (27%)*	12/30 (40%)	3/23 (13%)
Delayed white matter myelination	1/11 (9%)	9/30 (30%)	NR
Corpus callosum (CC) abnormalities	6/11 (54%)^†^	20/30 (67%)^‡^	7/23 (30%)^§^
Cerebellar abnormalities	3/11 (27%)^¶^	10/30 (33%)^¶#^	5/23 (22%)^¶^
Cerebral atrophy	8/11 (73%)	NR	6/23 (26%)
Brain stem atrophy	2/11 (8%)	NR	NR
Persistence of the cavum septum pellucidum	NR	12/30 (40%)	5/23 (22%)
Dilatated lateral ventricles	4/11 (36%)	9/30 (30%)	NR
Dilatated Robin-Virchow spaces	1/11 (9%)	1/30 (3%)	2/23 (9%)

*“White matter change”

^†^CC hypoplasia (6/11)

^‡^CC dysgenesis (20/30), CC hypoplasia (12/30), short but complete CC (8/30), defective modelling of the genu (2/30), partial agenesis of CC (1/30), complete agenesis (1/30)

^§^Unspecified

^¶^Loss/atrophy of cerebellar volume

^#^Vermis hypoplasia (6/30), subnormal height of the vermis (4/30)

Non-specific neuroradiological abnormalities have been found in most patients that have undergone brain imaging, although no specific brain malformation pattern has been identified (Table 13). The most extensive neuroradiological study to date was a 2016 French case series in which 28/30 patients had abnormal brain MRI findings [65]. Two-thirds (n = 20) of the patients had corpus callosum dysgenesis including hypoplasia (12/30) or short but complete corpus callosum (8/30); as well as reduced white matter volume in 12/30 patients, physiological delay of white matter myelination in 9/30 patients, persistence of the cavum septum pellucidum in 12/30 patients, dilatation of lateral ventricles in 9/30 patients and cerebellar abnormalities in 10/30 patients including vermis hypoplasia (6/30), subnormal height of the vermis (4/30) and cerebellar atrophy (3/30).

A summary of findings in other studies include cerebral atrophy [1, 3, 4, 10, 17, 19, 27, 38, 40, 63, 65, 68, 74, 81, 84], cerebellar atrophy [10, 79] (specifically vermis hypoplasia in some cases [17, 19, 30, 40, 61, 64, 65, 70]), cortical atrophy [2, 17, 19, 20, 25, 52, 71] delayed/impaired myelination [2, 16, 17, 32, 59, 65, 75, 79, 81], agenesis or hypoplasia of the CC [4, 13, 16–18, 25, 29, 30, 38, 40, 51, 56, 61, 64, 65, 70, 79, 81], enlarged cisterna magna [17, 21, 40], hydrocephalus [22, 32, 40, 56], gliosis [27], periventricular leukomalacia [17, 28, 71, 75], choroid plexus cysts [4, 56, 65], septum pellucidum cysts [2, 16, 25, 43, 61, 71], Dandy-Walker malformation/variant [13, 43, 64], unilateral/bilateral ventricular dilatation [2, 16, 17, 19, 25, 28, 30, 32, 40, 43, 45, 51, 52, 56, 64, 65, 69, 74], and reduction white matter hyperintensity [24, 28, 69].

### Other medical comorbidities (Table 14)

**Table 14:** Summary of other medical comorbidities found in patients with MDS reported in the literature; NR = not reported

Feature	Males n/N	Females n/N	Total n/N (%)	References
Anaemia	2/11	NR	2/11 (18%)	[32, 48]
Astigmatism	2/7	NR	2/7 (29%)	[40, 68]
Hearing loss	13/39	NR	13/39 (33%)	[1, 2, 8, 9, 32, 40, 77]
Hypermetropia	23/44	2/7	25/51 (49%)	[12, 25, 26, 74, 77]
Hyperbilirubinaemia	2/9	NR	2/9 (22%)	[16, 40]
Hypothyroidism	2/10	3/7	5/17 (29%)	[25, 32, 43, 53, 71]
Obesity	3/7	1/6	4/13 (31%)	[16, 25, 74]
Thrombocytopaenia	3/19	NR	3/19 (16%)	[17]

Visual problems have been reported in almost a half (n = 25) of 51 patients from five studies in the form of hypermetropia (Table 14), strabismus in just over two-thirds (51/73; Table 10) and astigmatism (2/7; Table 14), in keeping with the higher prevalence of refractive errors and ocular findings in children with syndromic ID [119]. This may suggest a need for ophthalmologic evaluation in children with MDS, but larger sample sizes are required in future studies and natural history data will be important in understanding the prevalence of hypermetropia by age group as this condition can improve over childhood [120]. In one case series [77], 7 of 23 patients were assessed as having mild-to-moderate hearing loss. The type of deafness was evaluated in only three cases, two of whom had conductive deafness in relation to recurrent otitis and one had perceptive deafness. Greater attention to sensory impairments among children with MDS is important for early management and to prevent further inhibition of language development and communication skills [121]. Less commonly reported medical comorbidities include anaemia [32, 48], hyperbilirubinaemia [16, 40], thrombocytopaenia [17], hypothyroidism [25, 32, 43, 53, 71] and obesity [16, 25, 74].

### Clinical differences between male and female patients

MDS was initially thought to be 100% penetrant in males and asymptomatic in carrier females, however case series since the mid 2000s started recording females with variable disease characteristics [9–11]. Since then, approximately 70 female patients with MDS have been reported in the literature [5, 9–11, 26–28, 33, 35, 41, 43, 51–56, 62, 65, 69–71, 74–76, 78, 79, 83, 113]. In a recent genotype–phenotype study [43 males, 5 females] clinical severity scores were worse in males than females as were the results of motor behavioural assessment [78]. Whilst it is understood that there is clinical variability within MDS, factors underlying variable expressivity between affected males and females have only partially been explored.

Possible pathogenetic molecular mechanisms in affected females could include (1) an unbalanced translocation between the X chromosome and an autosome [9, 11, 26, 27, 43, 51, 65, 70], (2) random XCI [70, 74, 83] and (3) skewed XCI in which the non-duplication carrying X chromosome is preferentially inactivated [28, 33, 35, 41, 43, 52, 54, 74]. To date, the prevailing hypothesis for asymptomatic females is the presence of skewed XCI with preferential inactivation of the X chromosome with the duplication [52, 65] although skewed XCI is not always detected. This may relate to variabilities in XCI in different tissues within an individual or may suggest that the are other modifying factors [122].

### Life expectancy

In the absence of any population-based prospective studies information about life expectancy in MDS is sparse. Within the French series of 86 male patients with an intrachromosomal *MECP2* duplication, 27% (n = 23) had died before 25-years of age in contrast to 39% (n = 34) of 88 patients in the literature in an earlier review [30]. Reported causes of death in MDS have included (a) repeated seizure events [8, 77] (or status epilepticus [16, 69]); (b) respiratory events [83] including recurrent respiratory infections [2, 11, 36, 70, 77] described as pneumonia [8, 13, 17, 48, 52, 61, 62, 75] (often caused by aspiration), bronchitis [36, 48], respiratory insufficiency [1, 36, 48] (secondary to infections [16]), and pulmonary hypertension [5, 65, 77]; and (c) non-respiratory-related infections including gastroenteritis [61], myocarditis [75], central nervous system infection [2, 75], and sepsis [5, 68]. Other patients have died from systematic inflammatory response syndrome [48], and cardiac failure [17, 48]. In summary, the causes of death reported in the literature highlight the severe burden of seizures and respiratory-health issues in MDS.

### Cortisol profile

The cortisol profile in MDS has been of interest as a potential biomarker of clinical severity. A tentative link between recurrent respiratory infections in MDS due to immune dysregulation/chronic inflammation and the hypothalamus pituitary-adrenal (HPA) axis has been made—as HPA-dysfunction has been noted in immune disorders such as systemic lupus erythematosus and Sjögren’s syndrome which exhibit *MECP2* overexpression [123]. In a recent study, a declining cortisol awakening response (CAR) in 17/31 patients was associated with a larger duplication size, increased number of hospitalisations for infections and increased severity as assessed by the Clinical Severity Scale (CSS) [78] designed for RTT [123]. If this potential biomarker is validated through further study, it may have utility for stratification in future clinical trials.

### Immunological profile

It has been suggested that duplication of *IRAK1*, which is involved in mediating proinflammatory immune responses in Toll-like receptor (TLR)/IL-1R signalling pathways [124], may contribute to the occurrence of recurrent infections in MDS [16, 18, 29, 46], and susceptibility to pyogenic bacteria [18]. There is a potential role for immunisation regimens for polysaccharide-encapsulated bacteria, which is supported by limited findings in three male patients who required booster shots for poor responses to vaccines against polysaccharide-encapsulated species such as *Streptococcus pneumoniae* and *Haemophilus influenzae* type B [13, 125]. Early studies did not find strong evidence of abnormal T- and B-cell numbers or serum immunoglobulin (IgM, IgE, IgA, IgD and IgG with subclasses) levels, or T-cell functional and complement activity study results that could have explained the increased susceptibility of infections [8, 13, 18]. Moreover, cases with a duplicated region inclusive of *IRAK1* with no history of respiratory infections have been reported [42, 53, 55], as has a female patient who had recurrent respiratory infections but not a duplicated *IRAK1* region [43]. Mice models of MDS with an overexpression of human *MECP2* without *IRAK1* have also been shown to be immunodeficient suggesting that immune defects in MDS may likely be independent of *IRAK1* duplication [126, 127].

A seminal study by Bauer et al. in 2015 investigated and expanded upon the infectious and immunologic phenotype of MDS [62]. Of import, six of 21 patients had an IgG_2_ deficiency—four of whom had an additional IgA deficiency. An additional three patients had low levels of IgG_2_. Reduced or low levels of IgG have subsequently been found in an additional four of 51 individuals from 10 studies [8, 13, 18, 19, 36, 61, 71, 73, 81, 84] and reduced or low levels of IgA in an additional 10 of 51 individuals from nine studies [8, 13, 18, 36, 38, 71, 73, 81, 84].

### Genotype–phenotype relationships

To date, no comprehensive genotype–phenotype association studies have been conducted in regard to the genes involved in the Xq duplication involving *MECP2*. The minimally duplicated region in MDS includes the *MECP2* and *IRAK1* genes [4, 14, 19, 25, 28, 30, 40, 42, 44, 49, 52, 58, 112], and individuals with a larger, cytogenetically visible Xq28 duplication have been reported to display characteristics such as microcephaly, pre- and post-natal growth deficiency, inguinal hernia, palate clefting and hypoplastic genitalia [11]. As a result, other genes involved in the duplication may be dosage sensitive and exert an effect. The few reported individuals with a triplication of *MECP2* have displayed a more severe phenotype [14, 33, 36, 49, 50, 71].

It has been suggested that Xq28 duplications involving the ID-associated filamin A gene (*FLNA*; 300017) contribute to the presence of chronic constipation [17], as *FLNA* point mutations have been found in families with pseudointestinal obstruction [128, 129]. Against this hypothesis is that constipation was not a feature in recent case studies on patients harbouring a Xq28 duplication involving *FLNA* without *MECP2* [42, 63, 130, 131]. The presence of Hirschsprung disease in MDS, although rare, has also been suggested to be linked to dysregulated levels of the *L1CAM* gene (which can be implicated in Xq28 duplications) as *L1CAM* mutations have been detected in patients with X-linked hydrocephalus with Hirschsprung disease [22, 132, 133]. Finally, duplications involving the ID-associated GDP dissociation inhibitor 1 gene (*GDI1*; OMIM 300104) have been noted to be associated with the presence of microcephaly in individuals with MDS in various case series [2, 14, 16, 19]. It has been suggested that microduplications involving *GDI1* are dose-dependent and are associated with more severe clinical phenotypes, including a Dandy-Walker malformation [134]. However, deeper genotype–phenotype relationship studies are required to support these associations in the context of *MECP2* duplication.

Unsurprisingly, a recent study found that a larger duplication size was correlated with greater clinical severity as measured using the CSS and Motor Behavioural Assessment Scale (MBA) [78]. Presence of the ID-related Ras-associated protein Rab-39B (*RAB39B*; OMIM 300774) gene in the duplication was also associated with greater clinical severity, as it was to a lesser extent when the duplication involved the *L1CAM* gene. Interestingly, microduplications in the Xq28 region including *RAB39B* but terminal to *MECP2* in males have been noted to manifest with cognitive deficits, behavioural abnormalities including hyperactivity and aggressiveness, and dysmorphic features [135, 136]. Mutations in *RAB39B* have also been found in patients with XLID displaying autism, epilepsy and macrocephaly [137].

It has been suggested that the regulation of *MECP2* gene dosage is important for neurotypical development as overexpressed levels of *MECP2* in peripheral leukocytes have been found in some children with autism [138]. In contrast reduced levels of MeCP2 protein have been found post-mortem in the frontal cortex of individuals with Rett syndrome (9/9) and autism (11/14) [139].

## Discussion

Clinical research in MDS over the past two decades has provided a foundation for the further characterisation of this rare, neurodevelopmental disorder. To date, a few large case series have provided detailed phenotypic information on MDS: (1) a 2011 review of all prior case studies/small series as well as the inclusion of 15 unreported males [30], (2) our 2017 series on 49 males and 7 females [76], (3) a 2018 French series on 59 males [77], and (4) a 2019 US series on 43 males and 5 females [78]. These four main publications have reported on different aspects of the disorder with overlap of the main medical comorbidities such as recurrent respiratory infections and seizures. As a result, no previous series encompassed the entire clinical history of the individuals described which may mean that disease burden is not fully reflected. Attempting to expand documentation of the phenotype, our comprehensive review has collated the findings from all published studies and series on individuals with MDS to date.

This review details the following features of MDS that are frequently described in the literature: intellectual disability, global developmental delay, regression, seizures, lower respiratory tract infections (LRTI), gastrointestinal problems, symptoms of autism, dysmorphic features, sleep disturbances and abnormal neuroradiological findings. This review also highlights areas that have received limited attention such as non-LRTIs (e.g., pharyngitis, tonsilitis, otitis, urinary tract infections), cardiovascular defects, urogenital abnormalities beyond cryptorchidism, behaviour or mood disturbances (other than autism), and autonomic dysfunction. Multiple health and developmental features can co-exist within a very broad phenotype and contribute to disease burden.

Where it is known that the same individual has been reported in multiple studies, care has been taken to eliminate replicates when calculating proportions (“Appendix”). Additionally, where it was suspected that individuals were likely to be replicated in separate studies, they were removed entirely from the calculation of proportions. However, it is likely that due to the nature of deidentified data, it may not be possible to eliminate replicated cases entirely such that the quality of the data we have provided could be slightly compromised. Furthermore, the reviewed series represent cross-sectional studies and the proportion of individuals with a particular medical comorbidity may change with age. Where time-to-event analysis, a statistical method which accounts for the censoring of data to calculate the conditional probability of an event occurring [140], has been used to report the likelihood of ascertaining walking or developing seizures or scoliosis [76], these data have been presented.

One limitation of this review lies in the possibility that some conditions may have been more favoured than others by previous researchers. This was evident for the aforementioned features with limited attention, in where the aggregated proportions reflected a low denominator. The scant information available on them highlights the need for a complete dataset which can fully capture MDS and the burden of this disorder. Whilst these studies have provided important data—to date, there has been no longitudinal study on individuals with MDS.

Another reason for requiring better health information is to understand the differences and similarities between MDS and RTT, two disorders caused by either too much or too little MeCP2. This need has been partially addressed by one study which has compared the features of regression, seizures and clinical severity between MDS and RTT using data from the natural history study for RTT and related disorders (NCT03077308) [113]. However, it is apparent from this review that even deeper phenotyping of MDS is required to enrich the understanding of the disorder. A prerequisite to eagerly anticipated clinical trials, is an adequate knowledge of the natural history of this disorder, not yet available.

Future research would benefit from the ascertainment of a much larger sample size and the collection of longitudinal data to better understand the complete disease phenotype including those features which have been given less focus. This would allow for use of validated scales to assess, for example, sleep and quality of life [141, 142]. Disease severity and assessment of comorbidities have previously been measured with severity scales such as the Clinical Severity Scale (CSS) and Motor Behavioural Assessment (MBA) developed for use in RTT [78, 113, 123]. For example, the CSS includes items for head/somatic growth and hyperventilation, which do not appear to be of major clinical concern to MDS [143]. Furthermore, the CSS assesses age of onset of regression which is a key feature of RTT and not of MDS [143]. The CSS also fails to capture the recurrent infections in MDS which appear to be a cardinal feature. The development and validation of an MDS-specific severity scale and other more relevant outcome measures would be beneficial in assessing the overall clinical burden of this disorder and for use in the conduct of clinical trials.

We propose that the limitations of this review and the lack of complete datasets in the literature be addressed through the development of an international rare disease database for MDS. As was the case for establishing rare disease databases such as the Australian Rett Syndrome Database (ARSD) in 1993 [144], the International Rett Syndrome Database (InterRett) in 2003 [145] and the International CDKL5 Disorder Database (ICDD) in 2012 [146], there is need for a similar registry for MDS as it is an ultrarare disorder with a low birth prevalence [5]. This effort could be guided by consumers and stakeholders to craft an agenda for MDS which will connect families of affected patients and clinicians with the research community. An international database encompassing a larger population will allow for the centralised collection of stronger and more generalizable data including the more poorly addressed aspects of this disorder. This should lead to an increased understanding of the natural history, genotypic and phenotypic heterogeneity and disease burden of MDS with better granularity. Such data may potentially reveal clinical endpoints that can hopefully be used to inform therapeutic developments. In facing the complex needs of children with MDS, it is important to understand the many domains of health affected in this disorder so that therapies and interventions can be adequately tailored. Such insight will require the concerted efforts of not only researchers but also stakeholders in the years to come.

## Data Availability

Not applicable.

## Appendix

- In Pascual-Alonso et al. [83], patient 1 was previously described in Mayo et al. [33] and patient 7 was described in Madrigal et al. [15] and Bijlsma et al. [43].
- Both studies by Peters et al. [49] featured 10 and 17 individuals respectively. As both study populations had an age range of 3–10 years, only the latter paper with 17 individuals was included in estimates.
- In Yi et al. [69], four patients were previously reported.
- In Yamamoto et al. [58], 11 patients are described but only four are new to the literature (patients 3, 5, 8 and 11).
- In Ramocki et al. [4] one male was previously reported but it is not clear which male individual—nonetheless one individual was removed from estimates.
- In Clayton-Smith et al. [17], three males were previously reported.
